# Antibody-dependent cellular cytotoxicity-mediating antibodies from an HIV-1 vaccine efficacy trial preferentially use the VH1 gene family

**DOI:** 10.1186/1742-4690-9-S2-P78

**Published:** 2012-09-13

**Authors:** M Bonsignori, J Pollara, MA Moody, TB Kepler, X Chen, TC Gurley, DM Kozink, DJ Marshall, JF Whitesides, J Kaewkungwal, S Nitayaphan, P Pitisuttithum, S Rerks-Ngarm, JH Kim, NL Michael, DC Montefiori, H Liao, G Ferrari, BF Haynes

**Affiliations:** 1 Duke University Medical Center, Durham, NC, USA; 2 Boston University School of Medicine, Boston, MA, USA; 3 Tropical Hygiene, Mahidol University, Bangkok, Thailand; 4 Armed Forces Research Institute of Medical Sciences, Bangkok, Thailand; 5 Clinical Tropical Medicine, Mahidol University, Bangkok, Thailand; 6 Department of Disease Control, Ministry of Public Health, Nonthaburi, Thailand; 7 US Military HIV Research Program, Rockville, MD, USA

## Background

The ALVAC-HIV/AIDSVAX-B/E RV144 vaccine efficacy trial showed an estimated efficacy of 31%. The immune correlates analysis raised the hypothesis that the observed protection in RV144 may be partially due to Antibody-Dependent Cellular Cytotoxicity (ADCC)-mediating antibodies in the presence of low levels of Env IgA antibodies. In this study we analyzed the Ig VH family usage of vaccine-induced ADCC mAbs isolated from memory B cells of vaccinees.

## Methods

From a total of 321,945 memory B-cells of 6 vaccinees we obtained 23 mAbs that mediated ADCC using IgG+ memory B-cell cultures (n=9) and Env-specific flow cytometric single memory B-cell sorting (n=14). ADCC activity was measured using both E.CM243 gp120-coated and E.CM235-infected target cells in a flow-based assay.

## Results

ADCC-mediating mAbs displayed a disproportionate usage of VH1 family genes (17/23; 74%), in particular the VH1-2 gene segment (10/17; 59%), as recently observed for CD4bs broadly neutralizing antibodies (HAAD bNAbs). In contrast, only 17.1% of 111 heavy chains isolated from cultures that did not mediate ADCC used the VH1 gene. VH1 ADCC-mediating mAbs showed a high degree of V(D)J amino acid similarity to both the VH (68-84%) and VL (70-87%) HAAD motifs. V(D)J rearrangements displayed modest levels of affinity maturation (0.5-5.1% for heavy chains and 0.4-4.3% for light chains). While none of the VH1 ADCC-mediating mAbs was capable of mediating HIV-1 neutralization, the strength of their ADCC activity correlated with the levels of heavy chain somatic mutations (p=0.02). We produced the reverted unmutated ancestor antibodies of two VH1 ADCC-mediating mAbs: one bound to B.MN Env and both reacted against autoantigens.

## Conclusion

ADCC-mediating antibodies induced by the ALVAC-HIV/AIDSVAX-B/E vaccine underwent limited affinity maturation, and preferentially used VH1 gene segments which share the HAAD motif with CD4bs bNAbs. These observations raise the hypothesis that HIV-1 Env preferentially selects VH1 family usage for distinct subsets of antibodies with different functions.

